# Association Between Workplace Sexual Harassment Experiences and Disclosure Attitudes Among Iranian Nurses: A Cross‐Sectional Analysis

**DOI:** 10.1155/jonm/2356837

**Published:** 2026-09-07

**Authors:** Samaneh Behzadi Fard, Zahra Askari, Reyhaneh Farzadnia, Mahlagha Dehghan

**Affiliations:** ^1^ Aligoudarz School of Nursing, Lorestan University of Medical Sciences, Khorramabad, Iran, lums.ac.ir; ^2^ Department of Medical Emergencies, Sirjan School of Medical Sciences, Sirjan, Iran; ^3^ Shahid Beheshti University of Medical Sciences, Tehran, Iran, sbmu.ac.ir; ^4^ HIV/STI Surveillance Research Center, and WHO Collaborating Center for HIV Surveillance, Institute for Futures Studies in Health, Kerman University of Medical Sciences, Kerman, Iran, kmu.ac.ir

**Keywords:** cross-sectional study, disclosure, nurses, sexual harassment, workplace

## Abstract

**Introduction:**

Workplace sexual harassment (WSH) represents a significant global issue within the nursing profession. This study aimed to examine the association between nurses’ experiences of WSH and their attitudes toward disclosure.

**Methods:**

This cross‐sectional study was conducted in Kerman, the largest city in southeastern Iran. Of the 338 eligible nurses invited to participate, 330 completed the survey (response rate: 97.63%). The nurses completed a demographic questionnaire, the Nurses Sexual Harassment Scale (NSHS), and a previously developed and psychometrically validated questionnaire assessing nurses’ attitudes toward disclosing sexual harassment. Data were collected from April to October 2024 and analyzed using SPSS Version 26. Descriptive statistics were used to describe demographic characteristics and mean scores. Data were analyzed using descriptive statistics, an independent samples *t*‐test, ANOVA, and multiple linear regression analysis.

**Results:**

The mean score of nurses’ attitudes toward disclosing WSH was 47.62 ± 8.31. The mean WSH score was 14.46 ± 7.02, which was below the midpoint of the scale. Based on the descriptive analyses, the highest mean WSH scores were observed among company‐employed nurses, nurses working fixed shifts, those employed in pediatric wards, and nurses working in Hospital B. Higher mean disclosure attitude scores were observed among nurses working fixed shifts and those employed in Hospital *D*. In the adjusted linear regression model, higher WSH scores were positively associated with higher disclosure attitude scores (β = 0.540, *p* < 0.0001). Older age (β = −0.089, *p* = 0.033), female sex (β = −0.081, *p* = 0.046), rotating shift work (β = −0.156, *p* < 0.0001), and employment in psychiatric wards (β = −0.179, *p* < 0.0001) were independently associated with lower disclosure attitude scores, whereas employment in pediatric wards was independently associated with higher disclosure attitude scores (β = 0.130, *p* = 0.002).

**Implications for Nursing Management:**

Nurse managers should promote safe disclosure environments by establishing confidential reporting systems, preventing retaliation, providing education on reporting procedures, and offering targeted support for nurses working in clinical settings and shift patterns associated with lower disclosure‐attitude scores.

**Conclusion:**

WSH experiences, age, sex, work shift, and clinical ward were independently associated with nurses’ attitudes toward WSH disclosure. These findings highlight the importance of addressing both individual‐ and organizational‐level factors to foster a supportive reporting environment and strengthen workplace safety in healthcare settings.

## 1. Introduction

Workplace sexual harassment (WSH) refers to any unwelcome behavior of a sexual or gender‐specific nature that violates a person’s dignity. Such behavior may occur through words, images, gestures, or actions [1]. WSH in healthcare settings is a pervasive issue worldwide. Among healthcare workers, nurses are often recognized as particularly vulnerable to such misconduct due to their close interactions with patients, colleagues, and supervisors [2].

A systematic review reported that the prevalence of sexual harassment among female nurses ranged from 10% to 87.3%, with an overall prevalence of 43.15% [3]. On average, 53.4% of nurses (both male and female) experience sexual harassment during their careers [4]. In Iran, sexual harassment against nurses remains a critical yet underreported issue [5]. Such harassment significantly impacts their mental health, physical well‐being, and professional performance [3].

Studies have shown that sexual harassment leads to severe psychological trauma among nurses, including mental health disorders, feelings of shame, anger, fear, and insecurity, which in turn negatively affect their ability to provide quality care [6, 7]. Despite the profound consequences, the reporting rate of sexual harassment cases remains low, primarily due to multifaceted barriers such as fear of social stigma, organizational and legal obstacles, family pressures, and personal fears [8, 9].

In Iran, cultural and organizational factors may influence the prevalence of sexual harassment and nurses’ attitudes toward disclosing such incidents. Previous studies indicate that only 2%–15% of WSH cases are officially reported [10, 11]. This silence perpetuates the cycle of harassment and exacerbates its harmful effects on nurses and the healthcare system [9]. Understanding nurses’ attitudes toward disclosing sexual harassment in the workplace is essential for breaking this silence and creating safer work environments [12].

A nurse’s attitude toward disclosing WSH may be influenced by various factors, including trust in institutional reporting mechanisms, fear of professional repercussions, personal stigma, and the perceived seriousness of the incident [13].

In a study by Mohammed et al., the prevalence of sexual harassment among nurses in Ghana was reported at 43.6%, with only a small number of victims filing complaints [14].

In another study by Tang et al., 63% of operating room nurses experienced sexual harassment in the past 12 months, and among them, 47.8% remained silent after the incident. Insufficient legal knowledge among nurses regarding how to report WSH and the lack of organizational support from hospitals were cited as reasons for their silence. In this study, only 39.2% of hospitals had channels or methods for reporting sexual harassment, and only 30.7% had departments to address WSH [15].

In conservative societies like Iran, nurses (especially women) may refrain from reporting for reasons such as fear of blame, shame, concerns about damaging their professional/personal reputation, fear of retaliation, inadequate protection for victims, and legal and workplace policies that may further discourage reporting [16].

In the Iranian sociocultural context, concerns about social stigma, fear of damaging professional reputation, hierarchical workplace structures, and traditional gender norms may discourage nurses from disclosing WSH. These contextual factors underscore the importance of understanding attitudes toward disclosure among Iranian nurses [9, 17].

Despite the significance of this issue, only a limited number of studies have examined Iranian nurses’ attitudes toward disclosing WSH. By improving understanding of nurses’ attitudes toward disclosure, this research seeks to inform targeted interventions and organizational policies aimed at promoting disclosure and creating safer work environments. According to the Theory of Planned Behavior, attitudes toward a behavior are important determinants of behavioral intentions [18]. Therefore, understanding nurses’ attitudes toward disclosing WSH may provide insight into their perceptions of disclosure and help inform organizational strategies aimed at creating supportive reporting environments. Accordingly, this study sought to answer the following research question: What is the relationship between Iranian nurses’ experiences of WSH and their attitudes toward disclosing such incidents?

## 2. Method and Materials

### 2.1. Study Design and Setting

This cross‐sectional study examined the relationship between WSH and attitudes toward its disclosure among Iranian nurses. Data were collected in Kerman, the largest city in southeastern Iran, between April and October 2024. The study was conducted in four tertiary teaching hospitals affiliated with Kerman University of Medical Sciences, which serve as the major referral centers in southeastern Iran.

These hospitals provide a wide range of inpatient services, including intensive care, emergency, psychiatric, pediatric, medical, and surgical wards. The total study population of approximately 1700 nurses was determined from the official staffing records of the participating hospitals. Nurses from different clinical roles (staff nurses, head nurses, and supervisors) and hospital wards were included to obtain a more comprehensive representation of nursing staff across diverse clinical settings.

The present study represents a planned secondary analysis of a larger cross‐sectional dataset previously used for the development and psychometric validation of the Nurses’ Attitudes toward Workplace Sexual Harassment Disclosure Scale (NAWSHD‐S) [12]. The previous publication focused on instrument development and psychometric evaluation. The current study addresses a distinct research question by examining the association between nurses’ experiences of WSH and their attitudes toward disclosure. The findings reported here have not been reported in the previous psychometric publication.

### 2.2. Sampling and Sample Size

After obtaining the necessary permits, the researcher visited educational hospitals in Kerman, Iran. The study population consisted of all nurses working in four educational hospitals. Of the 338 distributed questionnaires, eight were excluded because they were incomplete, resulting in a final sample of 330 nurses. For the few missing item‐level responses among the retained questionnaires, median imputation was applied, consistent with the approach used in the previous validation study.

To estimate the sample size, we used Cochran’s formula for an infinite population (*Z* = 1.96, *d* = 0.055, *n* = 317). Based on the drop‐out rate, 338 questionnaires were distributed using convenience sampling. After removing the incomplete questionnaires, data from 330 participants were included in the final analysis. The response rate was 97.63%.

### 2.3. Measures

#### 2.3.1. Demographic Characteristics Information Form

The demographic characteristics information form included age, gender, marital status, education level, work experience, recruitment status, job position, wards, shift type, and hospital type.

#### 2.3.2. NAWSHD‐S

This scale was designed and psychometrically validated by the researchers to measure nurses’ attitudes toward disclosing WSH in the context of a PhD dissertation [12]. Exploratory factor analysis showed that the scale consists of 18 items and 4 subscales, which include (1) Concerns About Personality Consequences; (2) Concerns About Processes and Organizational Outcomes; (3) Tendency Toward Alternative Strategies; and (4) Ethical Beliefs About the Disclosure of WSH. The scale explained 54.27% of the total variance. It uses a 5‐point Likert scale ranging from 1 (*strongly agree*) to 5 (*strongly disagree*). Items 16, 17, and 18 are reverse‐scored.

The total score ranges from 18 to 90, with higher scores indicating a more positive attitude toward the disclosure of WSH. This scale demonstrated good reliability, with Cronbach’s alpha of 0.774, McDonald’s omega of 0.770, and an ICC of 0.802.

#### 2.3.3. Nurses Sexual Harassment Scale (NSHS)

The NSHS was selected because it assesses all types of sexual harassment among nurses. This scale was developed by Zeighami et al. as the first instrument to measure sexual harassment in Iranian nurses. The scale consists of 15 items and two components: “latent sexual harassment” (9 items) and “manifest sexual harassment” (6 items). These components explained 68.4% of the total variance. The instrument is rated on a 5‐point Likert scale ranging from 0 (*never*) to 4 (*always*). Scores range from 0 to 60, with higher scores indicating greater experience of sexual harassment. The scale also demonstrated good reliability, with Cronbach’s alpha of 0.94, McDonald’s omega of 0.94, and ICC = 0.92 [19].

### 2.4. Data Collection

After obtaining ethical approval from the Ethics Committee of Kerman University of Medical Sciences, the questionnaires were administered to nurses who met the study’s inclusion criteria. To minimize social desirability bias, participants were assured that their responses would remain anonymous and confidential and were informed that there were no right or wrong answers. A total of 338 questionnaires were distributed during data collection, which took place from April to October 2024.

After removing eight incomplete questionnaires, the final analysis included data from 330 participants. The inclusion criteria were being employed as a registered nurse in one of the participating teaching hospitals, having at least a bachelor’s degree, which is the minimum educational qualification required for registered nurses in Iranian teaching hospitals, and having at least 1 year of work experience. Nurses in all job positions were eligible for participation. The exclusion criteria included unwillingness to participate and questionnaires with more than 10% missing items.

### 2.5. Study Hypotheses

Based on the study objective, the following hypothesis was formulated:

 H1: There is a significant association between nurses’ experience of sexual harassment and their attitudes toward disclosing sexual harassment.
 H0: There is no significant association between nurses’ experiences of sexual harassment and their attitudes toward disclosing sexual harassment.

### 2.6. Data Analysis

Data analysis was performed using SPSS Version 26. A *p*‐value of less than 0.05 was considered statistically significant for rejecting the null hypothesis. Descriptive statistics were employed to summarize demographic characteristics and mean scores. All mean comparison tests were conducted two‐tailed. The statistical framework included independent‐samples *t*‐test, one‐way ANOVA, and linear regression. A backward elimination method was utilized for the linear regression modeling.

To incorporate categorical variables with more than two levels into the model, dummy coding was applied, where a value of 0 indicated the absence and 1 indicated the presence of the attribute. For the regression equation, “married,” “bachelor’s degree,” and “emergency department staff” were designated as reference groups and were consequently excluded from the model. Additionally, gender was dummy‐coded as 0 for males and 1 for females, while work shifts were coded as 0 for fixed shifts and 1 for rotating shifts. Because of collinearity between the hospital and clinical ward, the hospital was excluded from the final regression model. Re‐running the model after explicitly removing the hospital yielded the same results.

## 3. Results

The mean score of nurses’ attitudes toward disclosing WSH was 47.62 ± 8.31, whereas the mean WSH score was 14.46 ± 7.02, which was below the midpoint of the scale. The mean age of the participants was 34.27 years, and the mean duration of work experience was 9.91 years. Most participants were female (80%), married (68.18%), held a bachelor’s degree (79.4%), worked as staff nurses (91.2%), had permanent employment status (50.6%), and worked rotating shifts (89.09%) (Table 1).

**TABLE 1:** Demographic characteristics and their associations with attitude toward disclosure of WSH and sexual harassment (*n* = 330).

Demographic characteristics		No	Sexual harassment					Attitude toward disclosure of WSH				
			Mean ± SD	Median	IQR	Test statistic	*p*	Mean ± SD	Median	IQR	Test statistic	*p*
Gender	Male	66	13.83 ± 7.61	15.00	13.25	−0.811	0.418 Independent samples *t-*test	48.22 ± 9.85	48	14.00	0.559	0.578 Independent samples *t*‐test
	Female	264	14.61 ± 6.87	15.00	11.00			47.49 ± 7.88	48	9.75		

Marital status	Married	225	14.50 ± 6.91	14.00	11.00	0.509	0.667 One‐way ANOVA	47.53 ± 8.25	47	9.50	0.669	0.572 One‐way ANOVA
	Single	82	14.28 ± 7.18	16.00	11.00			47.41 ± 8.29	48	11.50		
	Divorced	18	13.72 ± 7.88	14.00	15.5			48.66 ± 9.38	47.5	12.5		
	Widow	5	18.00 ± 7.07	17.00	12.5			52.4 ± 7.50	55	11.50		

Age group (year)	23–33	155	14.85 ± 6.95	15.00	12.00	1.327	0.267 One‐way ANOVA	48.41 ± 8.90	48	12.00	1.267	0.283 One‐way ANOVA
	34–44	137	14.47 ± 7.13	15.00	11.00			46.93 ± 7.93	47	10.00		
	> 44	38	12.78 ± 5.29	12.50	10.25			47.05 ± 6.86	47	6.50		

Job position	Nurse	301	14.19 ± 7.00	14.00	12.00	2.499	0.084 One‐way ANOVA	47.41 ± 8.42	47	10.00	1.326	0.267 One‐way ANOVA
	Head nurse	23	17.47 ± 7.15	18.00	8.00			49.95 ± 6.35	51	9.00		
	Supervisor	6	16.00 ± 5.29	16.00	9.00			50.33 ± 8.23	54	11.50		

Job experience (year)	1 to 10	188	14.67 ± 6.84	15.00	11.00	1.842	0.106 One‐way ANOVA	48.07 ± 8.49	48	11.00	1.207	0.300 One‐way ANOVA
	11 to 20	122	14.60 ± 7.27	15.00	10.5			47.39 ± 7.95	47	10.00		
	20 to 30	20	11.55 ± 6.73	9.50	11.5			45.15 ± 8.46	45.5	11.00		

Occupational status	New graduate nurse	67	12.95 ± 6.96	12.00	12.00	3.007	0.019 One‐way ANOVA	47.28 ± 9.37	47	12.00	1.386	0.238 One‐way ANOVA
	Company‐employed	44	17.52 ± 7.01	19.00	11.75			49.86 ± 8.65	50	12.25		
	Contractual	12	15.08 ± 5.16	15.00	6.5			44.25 ± 9.25	47	18.00		
	Project‐based	40	14.07 ± 7.50	15.00	9.00			47.00 ± 8.13	48	8.75		
	Official	167	14.30 ± 6.78	14.00	12.00			47.60 ± 7.66	47	10.00		

Education	Bachelor	262	14.50 ± 6.86	15.00	11.0	1.765	0.173 One‐way ANOVA	47.99 ± 8.12	48	10.25	1.909	0.150 One‐way ANOVA
	MSc	59	13.66 ± 7.77	12.00	13.00			45.81 ± 8.62	46	11.00		
	PhD	9	18.33 ± 5.72	16.00	9.00			49.55 ± 10.46	47	9.00		

Shift	Fix	38	19.89 ± 5.40	20.00	7.00	6.360	< 0.0001 Independent samples *t*‐test	55.07 ± 6.24	54.5	7.50	6.192	< 0.0001 Independent samples *t*‐test
	Rotation	292	13.75 ± 6.90	13.00	11.00			46.67 ± 8.05	47	9.00		

Ward	Emergency	35	12.22 ± 5.97	12.00	10.00	8.404	< 0.0001 (Welch)	45.25 ± 4.83	45	7.00	11.420	< 0.0001 One‐way ANOVA
	Psychiatric	34	11.08 ± 6.79	9.50	10.5			56.23 ± 6.54	54.5	6.25		
	Intensive	77	14.11 ± 6.16	13.00	9.00			47.31 ± 7.34	47	10.5		
	Pediatric	30	20.26 ± 5.05	19.00	7.00			41.20 ± 5.46	41.5	8.50		
	Internal	74	15.33 ± 7.10	16.00	12.25			48.86 ± 8.78	49	10.25		
	Surgery	74	14.24 ± 7.48	14.50	12.5			47.23 ± 8.69	48	7.50		
	Nursing office	6	13.83 ± 9.23	13.50	18.00			49.33 ± 8.75	49.5	11.25		

Hospital	A	154	14.57 ± 7.30	15.00	13.00	3.902	0.009 One‐way ANOVA	47.83 ± 8.67	47.5	9.00	8.129	< 0.0001 One‐way ANOVA
	B	92	15.55 ± 6.80	16.00	10.00			41.41 ± 8.19	41.5	10.00		
	C	50	14.58 ± 5.98	15.00	8.25			48.42 ± 6.42	48	8.50		
	D	34	10.82 ± 6.81	9.00	9.75			49.19 ± 6.67	50	11.00		

*Note:* no = frequency, project‐based = similar to official status for 5 years, A = internal hospital, B = surgical hospital, C = internal surgical hospital (heart‐centered), D = psychiatric hospital.

Abbreviations: IQR = interquartile range, SD = standard deviation.

Table 1 summarizes the associations between nurses’ demographic and occupational characteristics, WSH, and attitudes toward WSH disclosure. Significant differences in WSH scores were observed according to occupational status (*p* = 0.019), work shift (*p* < 0.0001), clinical ward (*p* < 0.0001), and hospital type (*p* = 0.009). Similarly, attitudes toward WSH disclosure differed significantly according to work shift (*p* < 0.0001), clinical ward (*p* < 0.0001), and hospital type (*p* < 0.0001). No statistically significant differences were observed according to gender, marital status, age group, job position, years of work experience, or educational level (*p* > 0.05).

In the unadjusted descriptive analyses, the highest mean WSH scores were observed among company‐employed nurses (17.52 ± 7.01), nurses working fixed shifts (19.89 ± 5.40), those employed in pediatric wards (20.26 ± 5.05), and nurses working in Hospital B (15.55 ± 6.80). The lowest mean WSH score was observed among nurses working in Hospital D (10.82 ± 6.81). For attitudes toward WSH disclosure, the highest mean scores were observed among nurses working fixed shifts (55.07 ± 6.24), those employed in psychiatric wards (56.23 ± 6.54), and nurses working in Hospital D (49.19 ± 6.67), whereas the lowest mean scores were observed among nurses working in pediatric wards (41.20 ± 5.46) and Hospital B (41.41 ± 8.19). These descriptive findings represent unadjusted comparisons and should be interpreted separately from the multivariable regression analyses.

Variables showing statistically significant associations in the univariate analyses were entered into a multiple linear regression model using the backward elimination method. Categorical variables with more than two categories were entered as dummy variables. Higher WSH scores were independently associated with higher disclosure attitude scores (β = 0.540, *p* < 0.0001). Older age (β = −0.089, *p* = 0.033), female sex (β = −0.081, *p* = 0.046), rotating shift work (β = −0.156, *p* < 0.0001), and employment in psychiatric wards (β = −0.179, *p* < 0.0001) were independently associated with lower disclosure attitude scores, whereas employment in pediatric wards was independently associated with higher disclosure attitude scores (β = 0.130, *p* = 0.002).

The mean attitude score toward disclosure of WSH was 47.62 ± 8.31. The mean sexual harassment score among nurses was 14.46 ± 7.02, which was below the midpoint of the scale (Table 2).

**TABLE 2:** Attitude toward disclosure of WSH and nurse’s sexual harassment subscales.

Scales	Subscales	No	Mean	SD	Median	Minimum	Maximum
Attitude toward disclosure of WSH	Subscale 1: Concerns about personality consequences	330	13.19	4.22	13.00	6.00	27.00
	Subscale 2: Concerns about processes and organizational outcomes	330	10.01	3.18	10.00	5.00	24.00
	Subscale 3: Tendency toward alternative strategies	330	13.04	3.18	13.00	4.00	20.00
	Subscale 4: Ethical beliefs about the disclosure of WSH	330	11.36	2.24	12.00	3.00	15.00
	Total	330	47.62	8.31	48.00	20.00	75.00

Nurses sexual harassment	Subscale 1: Latent sexual harassment	330	11.47	5.21	11.00	2.00	25.00
	Subscale 2: Manifest sexual harassment	330	2.99	2.83	2.00	0.00	12.00
	Total	330	14.46	7.02	15.00	2.00	34.00

*Note:* no = frequency.

Abbreviation: SD, standard deviation.

The Durbin–Watson statistic is presented in Table 3 below to assess potential autocorrelation. Since the number 1.963 is very close to 2, we can say that there is no autocorrelation problem in this model, and the regression assumptions in this regard are valid.

**TABLE 3:** Durbin–Watson statistic to assess potential autocorrelation.

Model summary				
*R*	*R* square	Adjusted *R* square	Std. error of the estimate	Durbin–Watson
0.713	0.509	0.498	5.88307	1.963

Variables showing statistically significant associations in the univariate analyses were entered into a multiple linear regression model using the backward elimination method. Categorical variables with more than two categories were entered as dummy variables. Higher WSH scores were independently associated with higher disclosure attitude scores (β = 0.540, *p* < 0.0001). Older age (β = −0.089, *p* = 0.033), female sex (β = −0.081, *p* = 0.046), rotating shift work (β = −0.156, *p* < 0.0001), and employment in psychiatric wards (β = −0.179, *p* < 0.0001) were independently associated with lower disclosure attitude scores, whereas employment in pediatric wards was independently associated with higher disclosure attitude scores (β = 0.130, *p* = 0.002. (Table 4).

**TABLE 4:** Linear regression between demographic variables, nurse’s sexual harassment, and attitude toward disclosure of WSH along with regression coefficients.

	Unstandardized coefficients	Std. error	Standardized coefficients	*t*	*p*‐value	95.0% confidence interval for *B*		Collinearity statistics	
	B		Beta			Lower bound	Upper bound	Tolerance	VIF
(Constant)	47.132	2.456		19.187	< 0.0001	42.299	51.964		
Sexual harassment	0.638	0.051	0.540	12.581	< 0.0001	0.538	0.738	0.830	1.205
Age	−0.103	0.048	−0.089	−2.138	0.033	−0.197	−0.008	0.886	1.129
SEX	−1.679	0.838	−0.081	−2.004	0.046	−3.328	−0.031	0.934	1.071
Shift	−4.042	1.094	−0.156	−3.695	< 0.0001	−6.194	−1.890	0.860	1.163
Occupation status = contractual	−2.895	1.741	−0.065	−1.663	0.097	−6.320	0.530	0.988	1.013
Ward = psychiatric	−4.872	1.117	−0.179	−4.362	< 0.0001	−7.070	−2.675	0.910	1.099
Ward = pediatric	3.745	1.183	0.130	3.166	0.002	1.418	6.071	0.907	1.102

*Note:* a. Dependent variable: attitude toward disclosure of WSH.

## 4. Discussion

This study examined the relationship between WSH and attitudes toward disclosure among Iranian nurses. Overall, nurses reported relatively low WSH scores, and the mean disclosure‐attitude score was 47.62 ± 8.31. Higher WSH scores were independently associated with higher disclosure‐attitude scores, whereas older age, female sex, working rotating shifts, and employment in psychiatric wards were independently associated with lower disclosure‐attitude scores. These findings suggest that nurses’ attitudes toward disclosure are associated with a combination of individual characteristics and workplace‐related factors.

The mean WSH score in the present study was 14.46 ± 7.02. Comparisons of absolute WSH scores with previous studies should be interpreted cautiously because previous research has used different instruments, scoring systems, operational definitions, and study populations. Previous studies among nurses and other healthcare workers have nevertheless consistently identified workplace violence and sexual harassment as important occupational concerns, although the reported magnitude varies across settings. Such differences may reflect variations in sociocultural norms, organizational policies, sampling characteristics, reporting practices, and measurement approaches. Therefore, the WSH score observed in the present study should not be interpreted as directly higher or lower than scores obtained using different instruments. In addition, cultural and organizational factors that discourage disclosure of sensitive experiences may contribute to lower self‐reported experiences in some settings, including Iran, potentially reflecting underreporting rather than genuinely lower occurrence.

The mean attitude score toward WSH disclosure was 47.62 ± 8.31. Because no validated cutoff was available for categorizing disclosure attitudes as favorable or unfavorable, this score was interpreted descriptively. This finding differs from previous studies reporting lower disclosure tendencies among healthcare workers, where fear of retaliation, social stigma, confidentiality concerns, and lack of organizational support were identified as major barriers to reporting [2, 20–22]. The observed disclosure‐attitude scores may reflect differences in sociocultural context, institutional support, or awareness of workplace rights. However, disclosure‐attitude scores should not be interpreted as evidence of actual reporting behavior, as concerns regarding retaliation, confidentiality, and organizational responses may still influence nurses’ disclosure practices.

Among workplace characteristics, significant differences in WSH and disclosure attitudes were observed across work shifts, clinical wards, and hospital types in the univariate analyses. However, after adjustment for potential confounding variables, only work shift and clinical ward remained independently associated with disclosure attitudes, whereas hospital‐related differences were no longer statistically significant. This finding suggests that the apparent differences between hospitals may have been explained by underlying individual or workplace characteristics rather than hospital type itself, highlighting the importance of interpreting adjusted analyses alongside descriptive comparisons.

Specifically, nurses working rotating shifts demonstrated significantly lower disclosure attitude scores than those working fixed shifts. Likewise, although psychiatric nurses showed the highest unadjusted disclosure‐attitude scores in the descriptive analyses, the multivariable regression model identified employment in psychiatric wards as an independent predictor of lower disclosure attitudes after controlling for demographic and occupational characteristics. Conversely, employment in pediatric wards remained independently associated with higher disclosure‐attitude scores. This pattern suggests that the higher disclosure‐attitude scores observed in psychiatric wards in the descriptive analyses may have been influenced by differences in participant characteristics across wards rather than by ward type itself. These findings emphasize that crude comparisons and adjusted analyses may lead to different conclusions because of the influence of confounding variables and should therefore be interpreted together rather than in isolation.

The observed associations suggest that workplace characteristics may influence nurses’ attitudes toward disclosure WSH through organizational factors such as workload, organizational climate, managerial support, psychological safety, and trust in reporting systems. However, these mechanisms were not directly measured in the present study and should therefore be interpreted cautiously. Nevertheless, the findings highlight the potential role of organizational culture and nursing leadership in creating supportive environments that facilitate WSH disclosure [23, 24].

Previous studies have reported inconsistent findings regarding the influence of work environments on WSH and reporting behaviors. Algwaiz et al. found that nurses working night or rotating shifts reported WSH less frequently than day‐shift nurses, which the authors attributed to greater workplace isolation and reduced access to managerial and human resource support [25]. In contrast, Basfr et al. reported higher exposure to WSH among nurses working morning shifts than among those working evening shifts [26]. Ramacciati et al. observed that emergency nurses experienced higher risks of WSH and were less likely to report these incidents because of the demanding and stressful nature of emergency care environments. Although our adjusted analyses identified ward‐related differences in disclosure attitudes, the mechanisms underlying these associations are likely to differ across healthcare systems and should be interpreted within each study’s organizational, managerial, and sociocultural context [27].

Similarly, Zeighami et al. reported that Iranian nurses considered modifications in work conditions—including changing clinical departments, adjusting work schedules, and improving workload management—as important strategies for reducing WSH. Many participants described changing their work environment as an effective way to avoid repeated exposure to perpetrators and to facilitate psychological recovery following harassment experiences [28]. Collectively, these findings suggest that organizational interventions extending beyond individual‐level approaches may improve disclosure behaviors. Future qualitative and mixed‐methods studies are warranted to examine how organizational culture, leadership behaviors, confidential reporting mechanisms, and nurses’ perceptions of psychological safety shape disclosure decisions across different clinical settings.

In the present study, female nurses demonstrated significantly lower attitudes toward disclosing WSH than their male counterparts. This finding may reflect broader gender‐related inequalities within healthcare organizations and the unique challenges female nurses encounter when considering whether to report harassment. Within the Iranian sociocultural context, concerns about social judgment, victim‐blaming, damage to professional reputation, and fear of negative workplace consequences may contribute to the lower disclosure‐attitude scores observed among female participants. These sociocultural and organizational barriers may therefore contribute to the lower disclosure attitudes observed among female participants.

This finding is consistent with previous research identifying fear of retaliation and stigma as major barriers to reporting WSH, particularly among female nurses [29, 30]. Previous studies have further highlighted that concerns about inadequate organizational support, potential employment consequences, uncertainty about legal protection, internalized shame, and fear of not being believed can substantially reduce nurses’ attitudes toward disclosing harassment [5, 6, 17]. Similarly, Mohammed et al. found that many victims responded passively to WSH despite its considerable psychological and occupational consequences.

Some participants perceived harassment as an unavoidable aspect of their work, whereas others considered leaving their positions or intended to resign to avoid further victimization [31]. Collectively, these findings emphasize that disclosure decisions are influenced not only by individual experiences but also by organizational culture, managerial responsiveness, and nurses’ confidence that reporting will result in fair and supportive action.

Another important finding of the present study was that higher WSH scores were independently associated with higher disclosure‐attitude scores. This observation is consistent with previous evidence suggesting that individuals who experience WSH may become more aware of the importance of reporting or more willing to disclose such experiences because of increased recognition of their personal and professional consequences [2, 16]. Nevertheless, higher disclosure‐attitude scores do not necessarily translate into actual reporting behavior. Numerous organizational barriers—including fear of retaliation, limited confidence in reporting systems, concerns about confidentiality, and inadequate managerial support—may affect actual reporting behavior even when nurses hold higher attitudes toward disclosure. Consequently, healthcare organizations should avoid assuming that higher disclosure‐attitude scores alone will lead to increased reporting unless supportive organizational structures are simultaneously strengthened.

Taken together, the findings of this study highlight the critical role of nursing management in creating psychologically safe work environments that encourage disclosure of WSH. Nurse managers should establish confidential and easily accessible reporting mechanisms, adopt zero‐tolerance policies toward WSH, provide education regarding reporting procedures, ensure trauma‐informed responses to disclosures, and protect staff from retaliation following reporting. Particular attention may be warranted for nurses working rotating shifts and in clinical settings where lower disclosure‐attitude scores were observed. Implementing these strategies may strengthen nurses’ trust in reporting systems and contribute to safer, more supportive healthcare workplaces.

One limitation of this study was the relatively small number of male participants, as men were less willing to complete the questionnaires than women. This issue should be explored further in future studies involving Iranian male nurses. Furthermore, the cross‐sectional design prevents causal inferences between WSH and disclosure attitudes, and the use of self‐report questionnaires may have introduced recall and social desirability biases despite efforts to ensure anonymity and confidentiality. Convenience sampling may also have introduced selection bias, limiting the generalizability of the findings to the broader population of Iranian nurses.

Although the NAWSHD‐S demonstrated satisfactory psychometric properties, its relatively recent development may limit direct comparisons with studies using other instruments. Moreover, this study assessed nurses’ attitudes toward disclosure rather than their actual reporting behavior. Because attitudes do not always translate into behavior, the findings should be interpreted with appropriate caution. Given Iran’s cultural diversity, future multicenter studies using probability sampling across different regions of the country are warranted to improve the generalizability of the findings and to examine actual disclosure behaviors and reporting practices.

Despite these limitations, this study has several strengths, including the use of psychometrically validated instruments, a relatively large sample size, and a high response rate (97.63%), which enhance the credibility of the findings. In addition, the use of multivariable regression analysis allowed the identification of factors independently associated with attitudes toward WSH disclosure after adjustment for potential confounding variables, thereby strengthening the robustness of the findings.

## 5. Implications for Nursing Management

The findings of this study have potential implications for nursing management. Given the cross‐sectional design, these recommendations should be considered as implications rather than demonstrated intervention effects. Healthcare organizations may consider strengthening confidential reporting mechanisms, protection against retaliation, and education regarding WSH and reporting procedures. Particular attention may be warranted for nurses working rotating shifts and in clinical wards where lower disclosure‐attitude scores were observed. These strategies may help reduce barriers to disclosure, although their effectiveness was not directly evaluated in this study.

## 6. Conclusion

This study demonstrated that WSH experiences, age, sex, work shift, and clinical ward were independently associated with nurses’ attitudes toward WSH disclosure. Higher WSH scores were associated with higher disclosure‐attitude scores, whereas older age, female sex, rotating shift work, and employment in psychiatric wards were associated with lower disclosure‐attitude scores. These findings suggest that both individual and workplace‐related factors influence nurses’ attitudes toward disclosure WSH.

Strengthening supportive organizational cultures, promoting psychological safety, and implementing effective reporting systems may enhance disclosure and contribute to safer working environments for nurses. Future multicenter and qualitative studies are warranted to further investigate the organizational and cultural factors influencing disclosure behaviors and to improve the generalizability of these findings across different healthcare settings.

## Author Contributions

The author contributions were determined separately for each publication based on the actual contributions to that specific study. In the previous scale development and validation publication, Mahlagha Dehghan and Samaneh Behzadi Fard contributed to all stages of the study, including data collection and management, analysis, writing, and editing, while Reyhaneh Farzadiania and Zahra Askari contributed to the quantitative and qualitative analyses, provided critical feedback, and contributed to manuscript preparation. In the present study, Mahlagha Dehghan and Samaneh Behzadi Fard contributed to the study design, analysis, writing, and editing, while Zahra Askari and Reyhaneh Farzadnia contributed to data collection and manuscript preparation.

## Funding

The authors received no specific funding for this work.

## Ethics Statement

The present study represents a planned secondary analysis of data collected as part of a larger doctoral dissertation project. The original ethics approval and written informed consent covered analyses related to the objectives of the doctoral research project, including secondary analyses addressing different research questions using the same anonymized dataset. All analyses were conducted in accordance with the approved research protocol and without collecting additional participant data. The study protocol was approved by the Ethics Committee of Kerman University of Medical Sciences (ethics code: IR.KMU.REC.1402.138).

The study was conducted in accordance with the principles of the Declaration of Helsinki. Before data collection, participants received an information sheet explaining the study objectives, procedures, confidentiality issues, voluntary nature of participation, and their right to withdraw at any time without any consequences. Written informed consent was obtained from call participants prior to completing the questionnaires. Given the sensitive nature of WSH, participants were provided with contact information for psychological support services, and a brief debriefing regarding the purpose of the study was conducted after completion of the questionnaires.

## Consent

The authors have nothing to report.

## Conflicts of Interest

The authors declare no conflicts of interest.

## Data Availability

The datasets used for the current study are available from the corresponding author upon request.
